# Autocrine regulation of human sperm motility by tachykinins

**DOI:** 10.1186/1477-7827-8-104

**Published:** 2010-08-26

**Authors:** Francisco M Pinto, Cristina G Ravina, Nerea Subiran, Antonio Cejudo-Román, Manuel Fernández-Sánchez, Jon Irazusta, Nicolas Garrido, Luz Candenas

**Affiliations:** 1 Instituto de Investigaciones Químicas, CSIC, Avda. Americo Vespucio 49, 41092 Sevilla, Spain; 2 Instituto Valenciano de Infertilidad de Sevilla, Avenida Republica Argentina 58, 41011 Sevilla, Spain; 3 Department of Physiology, Faculty of Medicine and Dentistry, University of the Basque Country, 48940 Leioa, Bizkaia, Spain; 4 Instituto Valenciano de Infertilidad de Valencia, Plaza de la Policía Local 3, 46015 Valencia, Spain

## Abstract

**Background:**

We examined the presence and function of tachykinins and the tachykinin-degrading enzymes neprilysin (NEP) and neprilysin-2 (NEP2) in human spermatozoa.

**Methods:**

Freshly ejaculated semen was collected from forty-eight normozoospermic human donors. We analyzed the expression of substance P, neurokinin A, neurokinin B, hemokinin-1, NEP and NEP2 in sperm cells by reverse-transcriptase polymerase chain reaction (RT-PCR), western blot and immunocytochemistry assays and evaluated the effects of the neprilysin and neprilysin-2 inhibitor phosphoramidon on sperm motility in the absence and presence of tachykinin receptor-selective antagonists. Sperm motility was measured using WHO procedures or computer-assisted sperm analysis (CASA).

**Results:**

The mRNAs of the genes that encode substance P/neurokinin A (TAC1), neurokinin B (TAC3), hemokinin-1 (TAC4), neprilysin (MME) and neprilysin-2 (MMEL1) were expressed in human sperm. Immunocytochemistry studies revealed that tachykinin and neprilysin proteins were present in spermatozoa and show specific and differential distributions. Phosphoramidon increased sperm progressive motility and its effects were reduced in the presence of the tachykinin receptor antagonists SR140333 (NK1 receptor-selective) and SR48968 (NK2 receptor-selective) but unmodified in the presence of SR142801 (NK3 receptor-selective).

**Conclusion:**

These data show that tachykinins are present in human spermatozoa and participate in the regulation of sperm motility. Tachykinin activity is regulated, at least in part, by neprilysins.

## Background

There is now convincing evidence that tachykinins are involved in the regulation of reproductive function [1-8]. Recent data have demonstrated that tachykinin receptors are present in human sperm and are functionally active suggesting a role for the tachykinin system in the regulation of sperm function [9].

Mammalian tachykinins comprise a family of regulatory peptides including substance P (SP), neurokinin A (NKA), neurokinin B (NKB) and hemokinin-1 (HK-1) [10-15]. In humans, tachykinins are the products of three different genes. The *TAC1 *gene gives rise to four different mRNA splicing isoforms (α, β, γ and δ) that encode SP (α, β, γ and δ) and NKA (β and γ). The *TAC3 *gene encodes NKB. The *TAC4 *gene can also generate four distinct mRNAs, named α, β, γ and δ, all of which encode HK-1 [1,4,11,12]. Tachykinins effects are mediated by three receptors named NK_1_, NK_2 _and NK_3_, which, in humans, are encoded by the *TACR1*, *TACR2 *and *TACR3 *genes, respectively [15-19]. The NK_1 _receptor is activated preferentially by SP and HK-1, the NK_2 _receptor by NKA, and the NK_3 _receptor by NKB [15-19].

The neutral endopeptidase EC 3.4.24.11, also named enkephalinase or neprilysin (NEP) is the major peptidase that degrades tachykinins in most human tissues [8,20-23]. NEP also degrades other bioactive peptides such as enkephalins, angiotensins, endothelin-1, cholecystokinins and bradykinin [24-28]. The enzyme is expressed in human sperm [9,25-27] and its inhibition by thiorphan causes a change in sperm motility that is partially mediated by opioids [27]. In addition to classical NEP, a homologous enzyme was recently described and named neprilysin-2 (NEP2) [29]. Human NEP2 has much higher substrate specificity and only degrades tachykinins and angiotensin I with efficiency similar to NEP [28]. There are also important differences between enzyme sensitivity to the classical inhibitors, thiorphan and phosphoramidon. Thus thiorphan behaves as a selective NEP inhibitor while phosphoramidon inhibits both enzymes with almost equal potency [24,28]. NEP2 is expressed predominantly in the testis [29-31] and studies in mice deficient in NEP2 have shown that this enzyme is involved in sperm function and oocyte fertilization [31]. However, the role of NEP2 in human reproduction has not jet been established.

In the present study, we investigated the expression and cellular localization of tachykinins and the tachykinin-degrading enzymes NEP and NEP2 in human spermatozoa, analyzed the effects of the NEP and NEP2 inhibitor phosphoramidon on sperm motility, and determined whether endogenous tachykinins are involved in the responses observed after neprilysin inhibition.

## Methods

### Chemicals

SR140333, SR48968 and SR142801 were a generous gift from Sanofi Recherche (Montpellier, France). Phosphoramidon was from Sigma (St. Louis, MO, USA). Drugs were dissolved in distilled water (phosphoramidon) or absolute ethanol (tachykinin receptor antagonists) and diluted into sperm washing medium to appropriate concentrations.

### Semen samples and sperm preparation

Freshly ejaculated semen was collected from forty-eight healthy donors (18-35 years old) after 3-4 days of sexual abstinence. The study was approved by the Ethics Committee of Consejo Superior de Investigaciones Científicas (CSIC) and all donors gave written informed consent. The samples were allowed to liquefy at 37°C for 30 min and examined for concentration and motility following World Health Organization (WHO) guidelines [32]. Liquefied semen samples were washed with modified human tubal fluid (mHTF, Irvine Scientific, Santa Ana, CA, USA) supplemented with 0.5% bovine serum albumin (BSA) and processed as previously described [9]. Briefly, sperm suspensions were centrifuged through Spermgrad-125 (Vitrolife, Kungsbacka, Sweden), washed in mHTF, allowed to swim-up for 1 hour at 37°C and the supernatant carefully aspirated. Semen motility and concentration were visually re-examined and the concentration adjusted to 50 million per ml for subsequent experiments.

### RNA extraction and reverse transcription-polymerase chain reaction (RT-PCR)

Total RNA was extracted from sperm pools from 8 different donors using TriReagent (Sigma) and cDNA was synthesized using the Quantitect Reverse Transcription kit (Qiagen, Venlo, The Netherlands). Specific oligonucleotide primer pairs used to amplify *TAC1, TAC3*, *TAC4*, the α, β, γ and δ isoforms of *TAC1 *and *TAC4*, the genes that encode NEP (*MME*), NEP2 (*MMEL1*), β-actin (*ACTB*) acrosin (*ACR*) and *CD4 *are shown in Table 1 and were synthesized and purified by Sigma Genosys (Cambridge, UK). *ACTB *served as an internal control while *ACR *and *CD4 *were used to verify the presence of sperm cDNA and to exclude the presence of leukocyte contamination, respectively [33-35]. Gene expression was also analyzed in human testis and in a pool of cDNAs from twenty different human tissues (human total RNA master panel, BD Biosciences Clontech, Palo Alto, CA) used as a positive control of amplification.

**Table 1:** Sequences of forward (F) and reverse (R) primers of indicated target and reference genes

*Gene*	*Forward Primer*	*Reverse Primer*	*Amplicon size (bp)*
***TAC1***	ACTGTCCGTCGCAAAATCC	ACTGCTGAGGCTTGGGTCTC	212
***α/δTAC1***	GGAGCCCTTTGAGCATCTTC	CTTTCATAAGCCATTTTGTGAGAGA	168/123
***β/γTAC1***	GGAGCCCTTTGAGCATCTTC	TTCATAAGCCACAGAATTTAAAGCTC	220/175
***TAC3***	CCAGTGTGTGAGGGGAGCA	TCCAGAGATGAGTGGCTTTTGA	266
***TAC4***	GG TCTCTTCTCTGTGTCTCCTGTCCTC	CATTTATTGAGTGCCTACTGTGTGCT	246
**α*TAC4v1***	TGTGGCCTTGGAGGAAGG	ACTGCTGCTTGACACTGAGA	415
**α*TAC4v2***	GCCAAGGAGAAAAAAAGCAT	ACTGCTGCTTGACACTGAGA	292
**β-*TAC4***	GGAAGCGAGTGGGAGCAT	ACTGCTGCTTGACACTGAGA	290
**δ-*TAC4***	AGTGGGAGGCAGAGAGGAT	ACTGCTGCTTGACACTGAGA	223
**γ-*TAC4***	AAGGAGAAAAAAAGGCAGAGAG	ACTGCTGCTTGACACTGAGA	229
***MME***	AGCCTCTCGGTCCTTGTCCT	GGAGCTGGTCTCGGGAATG	219
***MMEL1***	TGGACATCTTGGAGGTGGTG	GGAGTTCTGGTCGTCGTTCC	164
***ACTB***	TCCCTGGAGAAGAGCTACGA	ATCTGCTGGAAGGTGGACAG	362
***ACR***	CCCTCCCATTTCGTGTGG	CACAAGTCCAGGTCGATGAGA	180
***CD4***	AGAAAGACGCAAGCCCAGAG	GCACCAGAAGCAAGTGCCTAA	127

Amplification was performed in PCR buffer containing 3 μl of cDNA reaction mixture, 2.5 mM MgCl_2_, 0.2 μM primers, 200 μM dNTP's and 1.5 U of DNA polymerase (Immolase, Bioline, London, UK). Cycling parameters were: 15 s at 94°C; 20 s at 60°C and 20 s at 72°C for 35 cycles. In some experiments, a 1/50 dilution of amplified cDNA was reamplified in the same PCR conditions and with the same primers. The PCR products were separated by gel electrophoresis on 2.5% agarose. The amplicon sizes were verified by comparison with a DNA size ladder and the identity of the products was established by sequence analysis.

### Indirect immunofluorescence

Sperm cells were washed, resuspended in phosphate-buffered saline (PBS) and smeared onto poly-L-lysine-coated slides. Spermatozoa were fixed in cold methanol (-20°C, 20 min) and incubated with 2% casein in PBS for 120 min to block non-specific sites. Test slides were incubated with a primary polyclonal antibody designed to recognize SP (sc-9758, dilution 1:200), NKB (sc-14109, dilution 1:200), HK-1 (sc-47439, dilution 1:500), NEP (sc-9149, dilution 1:200) from Santa Cruz Biotechnology (Santa Cruz, CA); NKA (T-4446, dilution 1:400), NKB (T-4450, dilution 1:400) from Peninsula Laboratories Inc. (San Carlos, CA) and NEP2 (HPA 007876, dilution 1:400, recommended by the supplier for immunocytochemistry and sc-104450, dilution 1:100, recommended for western blot) from Sigma and Santa Cruz, respectively. Primary antibodies were incubated overnight at 4°C diluted in PBS. The specificity of antibodies was assessed by bibliographic references or by pre-absorption with the corresponding immunogenic peptide. Negative control slides were not exposed to the primary antibody and were incubated with a) rabbit or goat IgG or b) PBS and processed in the same conditions as the test slides. Samples were incubated for 60 min with appropriate FITC-conjugated secondary antibodies (Santa Cruz), mounted using Prolong Gold antifade reagent (Invitrogen, Molecular Probes, Eugene, OR) and examined with an Olympus BX-51 fluorescence microscopy (Tokyo, Japan).

### Western blot analysis

Western blotting was used to assess the specificity of NEP and NEP2 antibodies and performed essentially as described previously [9]. Sperm cells were obtained as indicated above. Seminal plasma was obtained by centrifugation of semen samples at 12000 × *g *for 10 min. Total proteins from semen or seminal plasma samples were extracted by sonication in urea extraction buffer (1% w/v SDS, 9 M Urea, 1 mM EDTA, 0.7 M mercapto-ethanol, in 25 mM Tris-HCl, pH 6.8), boiled for 2 min and processed by the PAGEprep Advance kit (Pierce, Rockford, IL). Proteins were separated by electrophoresis on 10% SDS-PAGE gels, transferred to polyvinyldifluoride (PVDF) membranes and processed with the Amersham advance ECL kit (Amersham, Buckinghamshire, UK). Primary antibody dilution was 1:10000 and for the secondary antibody was 1:100000.

### Human sperm motility studies

Motility analysis was performed manually or using a computer-assisted sperm analysis (CASA) system (Sperm Class Analyzer, Microptic, Barcelona, Spain) essentially as described previously [9,33]. Aliquots of semen samples (5 μL) were placed into a Makler Counting Chamber (Sefi Medical Instruments Ltd., Haifa, Israel) and at least 200 sperm cells were evaluated at each incubation time by phase contrast microscopy. Sperm movement was graded following WHO guidelines [32] and defined as: *a*: rapid progressive motility; *b*: slow progressive motility; *c*: non-progressive motility and *d*: immotility. Progressive motility (*a*+*b*), non-progressive motility (*c*) and immotility (*d*) were measured as percentage of the total (*a*+*b*+*c*+*d*) that was considered as 100%.

Individual sperm samples were divided in several aliquots and each aliquot was untreated (time-matched paired controls) or treated with a single concentration of phosphoramidon (1 nM-1 μM) or the corresponding solvent. Sperm motility was measured 5 min before agent addition (initial value) and after 1, 15, 60, 120 and 240 min contact time periods. The effect of phosphoramidon (1 μM) or its solvent were also investigated in aliquots pretreated for 45 min with the tachykinin NK_1 _receptor-selective antagonist SR140333 (10 nM) [36], the NK_2 _receptor-selective antagonist SR48968 (10 nM) [37], the NK_3 _receptor-selective antagonist SR142801 (10 nM) [38], a cocktail of the three antagonists (10 nM each) or the corresponding solvent. A maximum of two drug concentrations, or the corresponding solvent volume, were tested on each aliquot. Values of sperm progressive motility, non-progressive motility and immotility were expressed as the positive or negative percentage increment in motility produced by the drug relative to the value observed at the same time in solvent-treated or time-matched paired controls (Δ sperm motility).

The effects of phosphoramidon on sperm kinetic parameters were analyzed by CASA, with settings according to instructions by the manufacturer. The measured kinetic parameters were curvilinear velocity (VCL); straight-line velocity (VSL), average path velocity (VAP); percent linearity (LIN = VSL/VCL × 100) and percent straightness (STR = VSL/VAP × 100).

### Statistical analysis

Values (means ± SEM) were obtained by pooling individual data and *n *indicates the number of experiments in samples from *n *different donors. Statistical analyses were made using Mann-Whitney's U (for comparison of mean ranks between two groups) or Kruskal-Wallis (to compare more than two groups) nonparametric tests. These procedures were undertaken using GRAPHPAD PRISM (version 5.0). *P *< 0.05 values were considered significant.

## Results

### mRNA expression of tachykinins and neprilysins in human sperm

**Figure 1:**
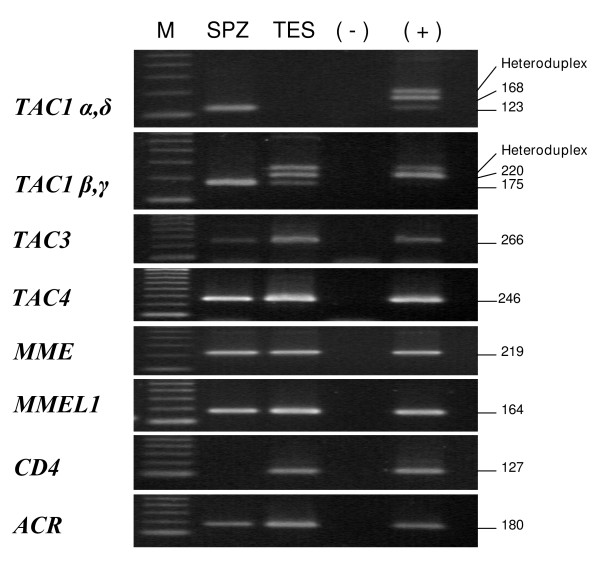
**Gene expression of tachykinin precursors, neprilysin (*MME*) and neprilysin-2 (*MMEL1*) in human sperm (SPZ) and testis (TES)**. In human sperm, the specific bands corresponding to *TAC1 *γ and δ isoforms, *TAC4 *and *MME *were only detected after cDNA reamplification. Acrosin (*ACR*) was present in sperm and testis cDNA while *CD4 *was only detected in testis. (+), positive control showing the expression of all genes in a pool of cDNAs from twenty different human tissues. (-), negative control with no RNA in the reverse transcriptase reaction; M, molecular size standards. The figure is representative of results in 6 pools of sperm samples from 8 different donors.

The genes that encode SP/NKA (*TAC1*), NKB (*TAC3*), hHK-1(*TAC4*), NEP (*MME*) and NEP2 (*MMEL1*) were detected in cDNAs from human sperm, testis and the pool of 20 human tissues used as positive control (Fig. 1). In sperm, the mRNAs of *TAC4 *and *MME *were only visualized after cDNA reamplification. Using specific primers and cDNA reamplification, we observed that, among the *TAC1 *isoforms, only the γ and δ transcripts were detectable in human spermatozoa (Fig. 1). *TAC1-*β, *TAC1-*γ and *TAC1-*δ were expressed in human testis, the last one being only observed after cDNA reamplification. Splice variants of the *TAC4 *gene were present in the positive control but were undetected in testis and sperm cDNAs (data not shown). We also verified the presence of *ACTB *and *ACR *and the absence of *CD4 *mRNA in all sperm samples (Fig. 1, not shown for *ACTB*).

Three negative controls were included in all assays (no reverse transcriptase, no RNA in the reverse transcriptase reaction and no template) and no PCR product was detected in any of these controls.

### Immunodetection of tachykinins and neprilysins in human sperm

**Figure 2:**
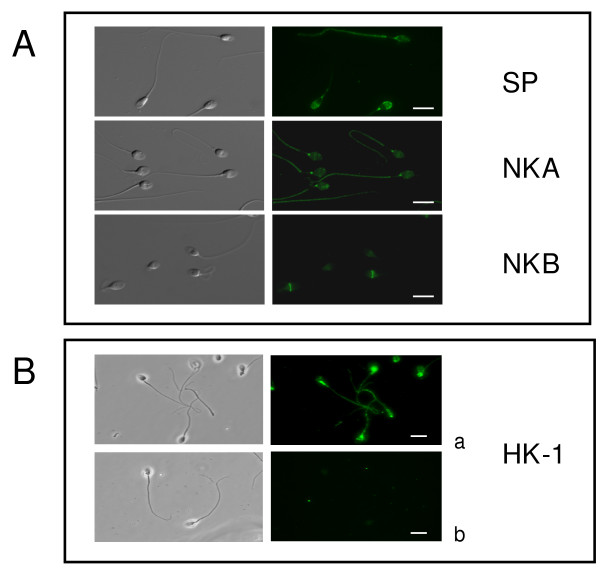
**Immunofluorescent localization of tachykinins in human sperm**. (A) Immunofluorescence and corresponding differential interference contrast images of sperm cells stained with primary antibodies against substance P (SP), neurokinin A (NKA) and neurokinin B (NKB) showing specific localizations for each peptide. (B) Immunofluorescence and corresponding phase contrast images of sperm cells stained with a primary antibody against hemokinin-1 (HK-1) in the absence (a) and in the presence (b) of immunogenic peptide. For each tachykinin, experiments were performed at least six times with similar results. Scale bar, 10 μM.

**Figure 3:**
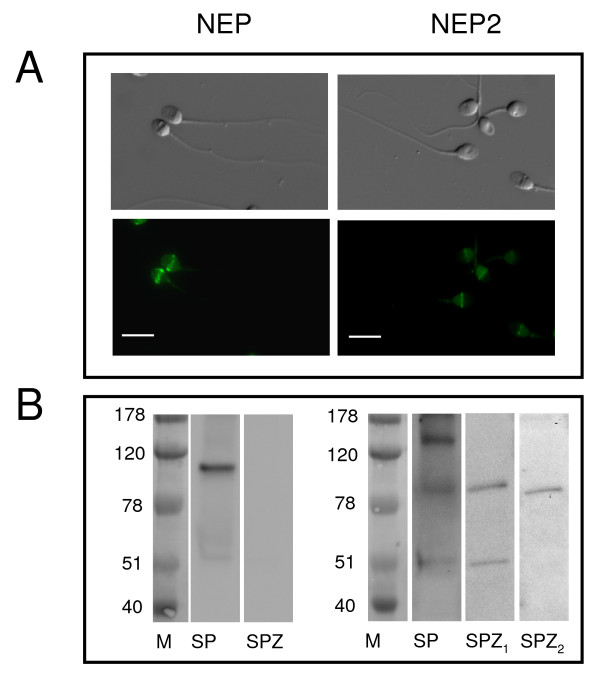
**Localization of neprilysins in human sperm**. (A) Immunofluorescence and corresponding differential interference contrast images of sperm cells stained with primary antibodies against neprilysin (NEP) and neprilysin-2 (NEP2). Experiments were performed at least six times with similar results. Scale bar, 10 μM. (B) Western Blot analysis of NEP and NEP2 in human spermatozoa (SPZ) and seminal plasma (SP) homogenates. For NEP2, SP and SPZ_1 _represent results obtained with the NEP2 antibody sc-104450 and SPZ_2 _represents results obtained with the NEP2 antibody HPA 007876 (see text for further details). Results are representative of at least five separate protein preparations, each from eight different donors.

**Figure 4:**
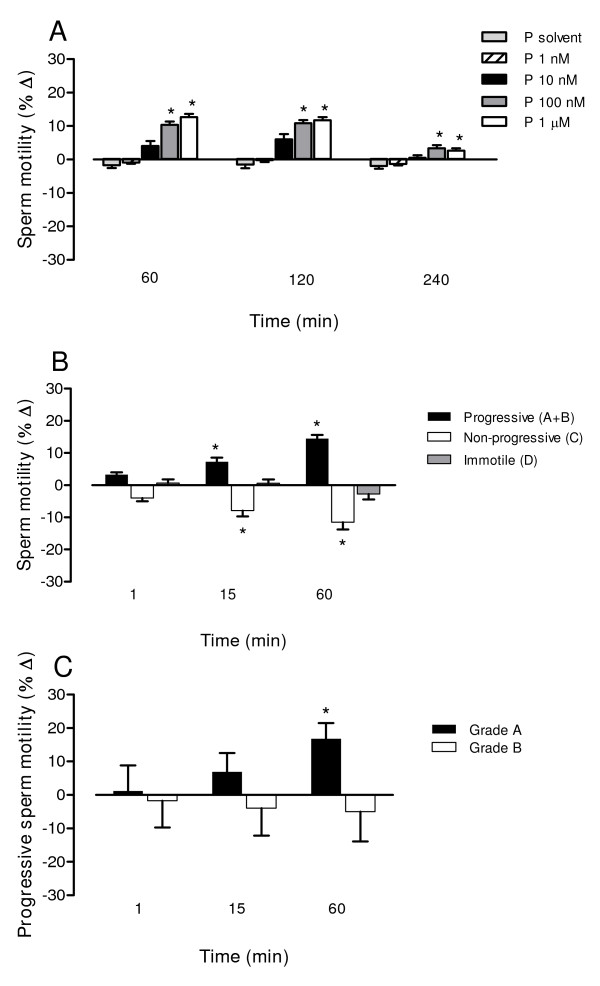
**Time- and concentration-dependent effects of phosphoramidon on human sperm motility**. Motility analysis was performed manually (A, B) or using a computer-assisted sperm analysis (CASA) system (C). (A) Effects of phosphoramidon (1 nM-1 μM) or its solvent on progressive motility (grade *a*+*b *sperm) at different times of incubation (B) Effects of phosphoramidon (1 μM) on progressive motility (grade *a*+*b *sperm), non-progressive motility (grade *c *sperm) and immotility (grade *d *sperm) at different times of incubation. (C) Effects of phosphoramidon (1 μM) on grade *a *and grade *b *sperm at different times of incubation. Bars are means with SEM of 6-13 different experiments and represent percentage changes in motility in samples treated with phosphoramidon relative to the value observed at the same time in untreated (A) or solvent-treated (B, C) paired controls. *P < 0.05, significant difference vs. control responses.

Immunocytochemistry demonstrated positive immunostaining for tachykinins, NEP and NEP2 in sperm cells (Figs. 2, 3 and 4). Intense SP labeling was observed over the acrosomal region and around the connecting piece in approximately 80% of the cells. In the other cells, SP immunostaining was only observed around the connecting piece and the flagellum principal piece (Fig. 2A). NKA was mainly found around the neck with a less intense immunostaining of the sperm head and the flagellum principal piece. NKB immunofluorescence was restricted to the equatorial segment and the post-equatorial region of the head (Fig. 2A). We found identical NKB immunolocalization with two different antibodies. HK-1 was present in the postacrosomal region and along the tail and intense labeling was detected in the flagellum midpiece being the only tachykinin that was present in this sperm area (Fig. 2B). Preincubation of the primary antiserum with SP or HK-1 immunogenic peptide (5 μg/ml) caused a disappearance of the fluorescent signal (Fig. 2B, not shown for SP). NEP immunostaining was localized in the equatorial segment in approximately 80% of cells, detected around the neck in a small, different population of sperm cells (approximately 3%) or undetectable (Fig. 3A). NEP2 was localized around the equatorial segment and the post-acrosomal region of spermatozoa with the HPA 007876 antibody (Fig. 3A). Unspecific binding was not observed in the presence of preimmune rabbit or goat serum and there was no immunofluorescence in the absence of the corresponding primary antibody.

Western blot confirmed the specificity of NEP and NEP2 antibodies and showed the presence of both enzymes in seminal plasma and of NEP2 in spermatozoa. The NEP antibody labeled a band of approximately 100 kDa (Fig. 3B). In agreement with previous data [27] the NEP signal was not observed in sperm. The NEP2 antibody HPA 007876, used in immunocytochemistry studies, recognized a band of approximately 90 kDa, the size expected for the major membrane-bound human NEP2 isoform [32]. As a positive control, we used a second human NEP2 antibody, sc-104450, recommended by the supplier for western blot. This second NEP2 antibody labeled the 90 kDa band in both spermatozoa and seminal plasma (Fig. 3B). In seminal plasma it recognized a second band, of approximately 130 kDa, which may correspond to an already described, additional membrane-associated isoform or to the soluble form of human NEP2 [32]. A third unknown band of approximately 50 kDa was detected with the sc-104450 antibody in seminal plasma and sperm cells. The immunoreactive bands for NEP and NEP2 were not observed when primary antibodies were omitted (data not shown).

### Effects of phosphoramidon on human sperm motility

The NEP and NEP2 inhibitor phosphoramidon caused time- and concentration-dependent increases in the proportion of progressively motile sperm (Fig. 4). The magnitude of the effect of phosphoramidon was inversely related to the initial proportion of progressive motile sperm in the sample. Thus, in samples with initial values of sperm progressive motility of 45-60%, the percentage of grade *a*+*b *sperm was 64.8 ± 3.8 in samples treated with 1 μM phosphoramidon for 60 min and 51.0 ± 3.2 in time-matched paired aliquots (*P *< 0.05). Sperm progressive motility was not affected by the phosphoramidon solvent (50.0 ± 3.3, *P *> 0.05 vs. time-matched controls). The results were similar when sperm motility was measured manually (Fig. 4A, B) or by CASA (Fig. 4C). The use of CASA revealed that phosphoramidon increased particularly the percentage of grade *a *spermatozoa (Fig. 4C). With regard to sperm kinetimatic parameters, CASA analysis showed that phosphoramidon increased straightness (by 6.13 ± 1.74%) and linearity (by 7.56 ± 0.98%) (*P *< 0.05 *vs*. solvent-treated aliquots) leading to a motility pattern characteristic of non-hyperactivated sperm.

**Figure 5:**
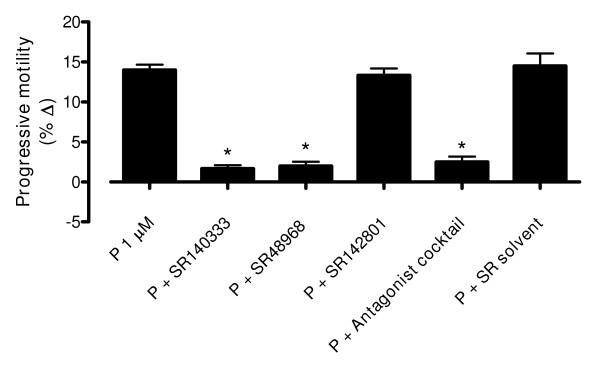
**Tachykinin receptor-selective antagonists inhibit the effect of phosphoramidon on sperm motility**. The effects of phosphoramidon (1 μM, 60 min incubation) on human sperm progressive motility (grade *a*+*b *sperm) were analyzed in the presence of SR140333 (NK_1 _antagonist, 10 nM), SR48968 (NK_2 _antagonist, 10 nM), SR142801 (NK_3 _antagonist, 10 nM), a combination of the three antagonists, or the antagonist solvent. Motility analysis was performed manually following WHO guidelines. Bars are means with SEM of 6-8 different experiments and represent percentage changes in motility in samples treated with phosphoramidon relative to the value observed at the same time in phosphoramidon solvent-treated paired controls. *P < 0.05, significant difference vs. response to phosphoramidon.

We then analyzed whether tachykinin receptors could mediate the actions of phosphoramidon on sperm motility. The effect of phosphoramidon (1 μM, 60 min incubation) was reduced by preincubation of the sperm sample for 45 min with the NK_1 _receptor-selective antagonist SR140333 (10 nM) or the NK_2 _receptor-selective antagonist SR48968 (10 nM) but was not affected by the NK_3 _receptor-selective antagonist SR142801 (10 nM) (Fig. 5). The phosphoramidon-induced effect was reduced, but not abolished, in the presence of a combination of the three tachykinin receptor antagonists (each at a concentration of 10 nM, Fig 5). The antagonist solvent had no effect on phosphoramidon responses (Fig 5).

## Discussion

In the present study, the major findings are: a) the tachykinin peptides, SP, NKA, NKB and HK-1 are present in human sperm; b) the two most important enzymes involved in tachykinin metabolism, NEP and NEP2 are expressed in human spermatozoa and c) the endogenous tachykinins modulate the motility of these cells.

The mRNAs of *TAC1*, *TAC3 *and *TAC4*, coding for the human tachykinin peptides, were expressed in sperm (9, this study). Among the *TAC1 *isoforms, only *TAC1-*γ and the rare *TAC1-*δ were present in spermatozoa. Conversely, the α and β isoforms were absent, in spite of the fact that *TAC1-*β is one of the most abundant *TAC1 *isoforms in many human cells and tissues including the testis [2,6,14]. With respect to *TAC4*, none of its splicing variants was detected. Increasing evidence suggests that the specific fraction of mRNAs that stays in mature spermatozoa plays some role in subsequent fertilization steps or is required for adequate embryo formation [39-42]. In this context, recent reports have established the existence of important differences between the sperm transcriptome of fertile and infertile men [42] providing clinical support for the relevance of sperm mRNA in male fertility.

The mRNAs of all tachykinins were present in human testis and sperm. Because spermatozoa are considered transcriptionally silent cells, the presence of these mRNA unequivocally proves that the genes encoding these proteins are transcribed in germ cells at any step during spermatogenesis. In addition, immunocytochemistry studies revealed that all tachykinin peptides were present in mature spermatozoa. Major labeling was observed over the acrosomal region for SP, around the connecting piece for NKA, in the equatorial/post-acrosomal region for NKB and around the midpiece for HK-1. This regional pattern of distribution argues for a specific role for each tachykinin in the regulation of sperm function. The presence of NKB in the equatorial segment suggests that this tachykinin could be involved in the fertilization process because this segment appears important in the fusion of gametes [43]. It has recently been shown that the NKB/NK_3 _ligand-receptor pair plays a central role in the regulation of reproductive functions [4-9]. In this context, it is interesting to note that NKB immunostaining was in a similar location than that previously found for the tachykinin NK_3 _receptor [9].

The local bioactivity of peptide signaling molecules is tightly controlled by their enzymatic degradation. Our data show that NEP and NEP2, the most important enzymes involved in tachykinin metabolism, are expressed in human sperm at both mRNA and protein levels. In reference to NEP, the data confirm previous results showing the expression of this enzyme in sperm [25-27]. With respect to NEP2, we report for the first time the presence of this enzyme in human spermatozoa. The observation that NEP2 was placed around the equatorial segment of human spermatozoa support a role for this enzyme in sperm fertilizing ability. These data are in line with previous findings showing that sperm from NEP2 knockout mice show apparently normal characteristics but lower oocyte fertilization and reduced embryo development [31].

NEP and NEP2 were abundant in seminal plasma suggesting that the activity of their substrates must be strictly controlled during the last maturation steps in the male reproductive tract and/or during ejaculation. In mature spermatozoa, inhibition of both enzymes by phosphoramidon caused an increase in sperm progressive motility. Sperm motility is an important feature and the most reliable actual predictor of male factor infertility [44,45]. Our data show that phosphoramidon induced a rise in straightness and linearity leading to motility trajectories that are characteristics of non hyperactivated spermatozoa and this is important because only spermatozoa with good progressive motility are able to swim through the female reproductive tract and reach the oviduct.

The responses elicited by phosphoramidon were reduced in the presence of SR140333, a selective antagonist of the tachykinin NK_1 _receptor [36], and in the presence of SR48968, a selective antagonist of the tachykinin NK_2 _receptor [37]. This demonstrates that the effects observed after neprilysin inhibition are mediated, at least in part, by tachykinins acting at the NK_1 _and the NK_2 _receptor. Conversely, the effects of phosphoramidon were unaffected in the presence of SR142801, a selective antagonist of the tachykinin NK_3 _receptor [38], consistent with our previous data which showed that the NK_3 _receptor plays a minor role in mediating motility changes induced by exogenously applied tachykinins in human sperm [9].

The effects of phosphoramidon were reduced, but not abolished, in the presence of a combination of SR140333, SR48968 and SR142801. NEP and NEP2 participate in degradation of other peptides, such as angiotensin-1, bradykinin and enkephalins which are also implicated in sperm cell function [27,28] and might thus be relevant to explain the tachykinin antagonist-resistant component of the response to phosphoramidon. In fact, the NEP inhibitor thiorphan increased sperm progressive motility and the opioid receptor antagonist naloxone inhibited the effects observed after prolonged (4 h) but not shorter (2 h) periods of incubation with thiorphan [27]. It thus seems that both tachykinins and opioids are involved in the responses observed after neprilysin inhibition in human sperm without ruling out the possible involvement of other peptide substrates.

Bioactive peptides i.e., opioids [27], bradykinin [28] or tachykinins [[9], this study] are widely expressed in sperm and many of the enzymes involved in their metabolism are also present and are functionally active [25,27,28]. Inhibition of these enzymes caused slowly developing changes in sperm motility [[27,28], this study]. Thus, these biopeptides, and particularly tachykinins, could operate as signal molecules between spermatozoa and their environment acting in an autocrine and/or paracrine (effect on other cells, on the female genital tract, and *viceversa*) fashion. In fact, NK_1 _NK_2 _and NK_3 _receptors are present in sperm and exogenously applied tachykinins modulate progressive motility at nanomolar concentrations [9]. Further studies will help to clarify the precise role of neprilysins and neprylisin-sensitive peptides in the regulation of sperm physiology and male fertility.

## Conclusions

The present study shows that tachykinins and the tachykinin-degrading enzymes NEP and NEP2 are present in human spermatozoa and participate in the regulation of sperm motility. These data support a role for the tachykinin system in the regulation of sperm function.

## Abbreviations

SP: substance P; NKA: neurokinin A; NKB: neurokinin B; HK-1: hemokinin-1; NEP: neprilysin; NEP2: neprilysin-2; BSA: bovine serum albumin; RT-PCR: reverse-transcriptase polymerase chain reaction; CASA: computer-assisted sperm analysis; WHO: World Health Organization; MHTF: modified human tubal fluid; PBS: phosphate-buffered saline.

## Competing interests

The authors declare that they have no competing interests.

## Authors' contributions

FMP carried out RT-PCR, western blot analysis and participated in the design of the study. NS and ACR carried out motility studies and immunofluorescence experiments. CGR and MFS participated in sample collection and analysis of sperm parameters. NG, JI and LC wrote the manuscript and participated in the design of the study. All authors read and approved the final manuscript.
